# The concentration data of heavy metals in Iranian grown and imported rice and human health hazard assessment

**DOI:** 10.1016/j.dib.2017.11.057

**Published:** 2017-11-21

**Authors:** Ali Jafari, Bahram Kamarehie, Mansour Ghaderpoori, Nahid Khoshnamvand, Mehdi Birjandi

**Affiliations:** a Department of Environmental Health Engineering, School of Health and Nutrition, Lorestan University of Medical Sciences Khorramabad, Iran; b Department of Biostatistics, School of Health and Nutrition, Lorestan University of medical sciences, Khorramabad, Iran

**Keywords:** Food safety, Heavy metals, Iran, Rice, Risk assessment

## Abstract

The aim of this study was to review the prevalence of rice contamination to heavy metals in Iranian grown and imported rice brands by conducting a systematic review and assess the related human health risk. Multiple keywords such as "rice, heavy metals, and Iran" were used to search in related databases. The average concentration of Cd, Pb, As, Cu, Zn, Cr, Ni and Co for Iranian grown/imported rice were calculated as 0.16±0.08/0.13±0.05, 0.196±0.16/0.55±0.56, 0.046±0.002/0.057±0.0035,0.29±0.05/0.61±0.31, 26.13±10.3/3.46±2.49, 0.22±0.04/0.76±0.101, 16±7.3/2.08±0.34 and 0.29±0.047/0.29±0.07 mg kg^−1^, respectively. Except Co, there were significant differences between Iranian and imported rice brands. Estimated weekly intake for none of the metals exceeds the provisional tolerable weekly intake value. Accordingly, the rice types consumed in Iran have no health hazard for consumers.

**Specifications Table**

**Table:** 

Subject area	Chemistry, Biology
More specific subject area	Describe narrower subject area
Type of data	Table, figure
How data was acquired	Data of this study have been extracted from previous studies in this field
Data format	Raw, analyzed,
Experimental factors	The average of Cd, Pb, As, Cu, Zn, Cr, Ni, and Co for Iranian grown/imported were calculated (based on previous studies). After determining the heavy metal concentration, human risk assessment and exposure to heavy metals through rice consumption was performed.
Experimental features	Determining of heavy metals in Iranian and imported rice
Data source location	City, Country and/or Latitude & Longitude (& GPS coordinates) for collected samples/data if applicable
Data accessibility	Data are included in this article and supplemented excel file

**Value of the data**

• Rice as a food ingredients is wildly used around the world especially in Iran. According to FAO reports, about 30% of total energy and 20% of the protein source of world population are provided through rice consumption.
• Recently, food contamination by heavy metals has been considered increasingly as a serious threat due to potential accumulation of in human body, plants, crops and animals finally enter to food chains
• In Iran, anciently, rice has been cultivated in some locations especially in north of the country, but due to high demand it is imported from other countries such as Thailand, India and Pakistan.
• Heavy metals such as lead, cadmium and arsenic are xenobiotic, in the sense that these elements are not required for the body metabolism even in trace amounts are potentially toxic for human
• Plants such as rice could accumulate heavy metals in the crop and transfer the metals to human body

## 1 Data

### 1.1 Experimental design, materials and methods

#### 1.1.1 Search strategy

**Fig. 1:**
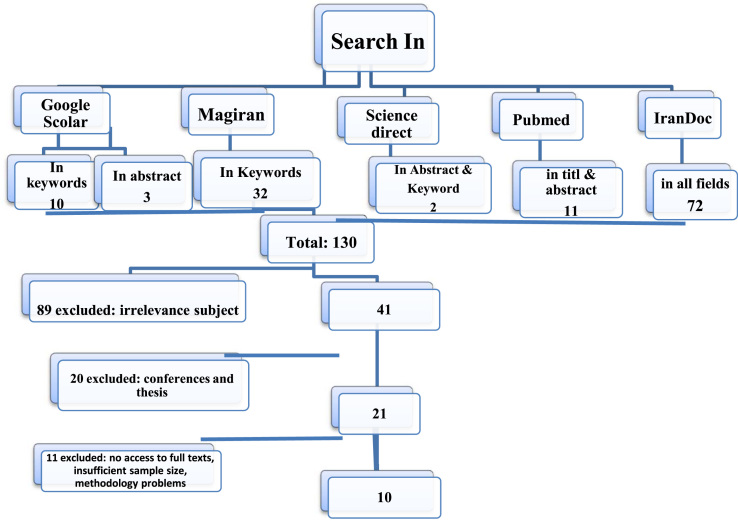
The view of search steps.

This systematic review was carried out based on published original articles in all publications (Internal and external databases, 2000–2017). In this review, internal (Persian Journals) and international databases such as Scientific Information Database (www.sid.ir), Magiran (www.Magiran.com), Iranian Research Institute for Information Science and Technology (www.irandoc.ac.ir), Iran Medex (www.iranmedex.com), and other famous English databases including Google scholar, Pubmed and Web of science were searched for key words of: “heavy metals”, “rice”, “Iran” (and Persian equivalents) in fields, title, abstract and keywords. Fig. 1 shows the search strategy diagram. After the first stage, the found articles were checked for eligibility for this review. Finally, the essential data was obtained through the selected articles and insert to spread sheet for further analysis.

#### 1.1.2 Search results

According to the initial search, totally 130 articles were found through the databases published in 2000–2017. By screening the titles, 89 works were excluded due to irrelevancy to the subject. At second screening stage, 20 theses and conference work works were also excluded (due to no access to full text). Furthermore, 11 other works were excluded due to lack of necessary data, including insufficient sample size and no access to full text. Finally, 10 articles were selected for the analysis. According to the obtained data, the most studied heavy metals in imported and domestic rice brands were cadmium, lead, arsenic, chromium, zinc, chromium, nickel and cobalt. For each metal the mean, standard deviation (SD) and pooled SD were calculated as presented in a Table 1, Table 2 for Iranian grown and imported rice brands, respectively.

**Table 1:** Driven data from published studies for heavy metals content in Iranian grown rice.

**Location**	**Sample size**	**Cd**	**Pb**	**As**	**Cr**	**Zn**	**Ni**	**Cu**	**Co**	
Kashan-2015	45	0.64±0.054	0.64±0.3	–	–	–	–	–		[1]
Mazandaran-2013	30	0.193±0.2	0.17±0.15	–	0.24±0.06	–	–	–	–	[2]
Kermanshah-2013	7	0.013±0.0007	0.275±0.003	0.046±0.002	–	–	–	–	–	[3]
Lorestan-2011	99	0.036±0.04	0.075±0.07	–	–	–	–	–	–	[4]
Shiraz-2010	15	0.34±0.06	0.243±0.06	–	0.39±0.03	–	0.76±0.101	–	0.29±0.05	[5]
Tehran-2015	45	–	–	–	–	20.7±0.31	–	1.1±0.03	–	[6]
Lorestan-2010	99	0.044±0.05	0.11±0.04			28.6±12.39		22.8±0.03		[7]

**Table 2:** Studies of heavy metals in imported rice.

**Location**	**Sample size**	**Cd**	**Pb**	**As**	**Cr**	**Zn**	**Ni**	**Cu**	**Co**	
All-2016	100	–	0.33±0.14	–	0.62±0.42	3.46±2.49	0.13±0.05	2.08±0.34	–	[8]
Kashan-2015	81	0.046±0.069	0.81±1.08	–	0.68±0.09	–	0.02±0.009	–	–	[1]
All-2011	60	–	0.37±0.12	–	–	–	–	–	–	[9]
Tabriz-2014	30	0.109±0.004	0.29±0.005	0.055±0.004	–	–	–	–	–	[10]
Kermanshah-2013	14	0.008±0.0001	0.216±0.002	0.052±0.002	–	–	–	–	–	[3]
Shiraz-2015	35	0.4±0.035	1.25±0.27	–	0.45±0.06	–	0.8±0.05	–	0.29± 0.07	[5]

### 1.2 Human Risk assessment and exposure to toxic metals

Human risk assessment and exposure to heavy metals through rice consumption was performed according to the procedure that has been widely used elsewhere. For this, the estimated weekly intakes (EWI), provisional tolerance weekly intake (PTWI) were calculated and compared with the values recommended by WHO/FAO expert committee. EWI was calculated by Eq. (1):

(1) $$EWI=C*(\;\frac{WC}{BW}\;)$$

where, EWI is estimated weekly intake (mg kg^−1^ bw week). C, WC, and BW are the mean of heavy metal content in rice (mg kg^-1^ dry weight), denotes the weekly rice consumption for each person (g week^−1^) (770 g per capita per week), and average body weight (kg), the average of Iranian grown body weight is 60 kg, respectively. Finally, the EWI was compared to PTWI provided by WHO/FAO. The mean concentrations of heavy metals for Iranian grown and imported rice types are represented in Table 3. For Cd, by comparing EWI and PTWI (Table 4) it was revealed that the EWI was 2.05 µg kg^−1^ BW and 1.69 µg kg^−1^. BW for Iranian grown and imported rice was lower than the recommended PTWI (7 µg kg^−1^ BW) provided by FAO/WHO, respectively. Hence, the consumption of the rice is not considered as a health risk factor in term of Cd. For Pb, statistical analysis revealed a significant difference between Iranian grown and imported rice and with the standard (*P*<0.001). But with regard to EWI and comparing with the provided value by WHO/FAO, the EWI values for both rice types are lower than PTWI. This metal intake at this level cannot be a risk according to PTWI. For As, its content in Iranian grown and imported rice were lower than Iranian standard and WHO guideline based on EWI. It is important to be noted that cooked rice may increase the heavy metal content to 1.642 mg kg^−1^. For Cr, There was a significant difference between Iranian grown and imported rice. From stand point of health hazard exposure, the values of EWI for Iranian cultivated and imported rice were 3.72 µg kg^−^^1^ BW and 7.83 µg kg^−1^ BW, respectively, comparing with reference value of PTWI (23.3 µg kg^−1^ BW the are safe and has no health hazard for consumers). For Ni, from stand point of health hazard the EWI value for Iranian and imported rice were 2.82 (µg kg^−1^ BW) and 9.75 (µg kg^−^^1^ BW), respectively, which was lower than PTWI (35 (µg kg^−1^ BW)). Hence, there is no hazard from the common consumption of rice intake by this metal.

**Table 3:** The mean concentration of heavy metals for Iranian grown and imported rice.

**Element**	**Type**	***N***	**mean (mg kg**^−^^**1**^**)**	**SD**	**DF**	***P***_**value**_
**Cd**	Iranian grown	295	0.16	± 0.08	0.03	p<0.001
	Imported	160	0.13	±0.05	–	p<0.001
**Pb**	Iranian grown	295	0.196	±0.16	-0.354	p<0.001
	Imported	320	0.55	±0.56	–	p<0.001
**As**	Iranian grown	7	0.046	±0.002	-0.011	p<0.001
	Imported	44	0.057	±0.0035	–	p<0.001
**Cr**	Iranian grown	45	0.29	±0.05	-0.32	p<0.001
	Imported	195	0.61	± 0.31	–	–
**Zn**	Iranian grown	144	26.13	±10.3	22.67	P = 0.99
	Imported	100	3.46	± 2.49	–	–
**Ni**	Iranian grown	195	0.22	± 0.04	-0.54	p<0.001
	Imported	15	0.76	±0.101	–	–
**Cu**	Iranian grown	166	16	±7.3	13.92	p<0.001
	Imported	100	2.08	± 0.34	–	–
**Co**	Iranian grown	15	0.29	±0.047	–	P=0.99
	Imported	30	0.29	±0.07	–	–

**Table 4:** Different permissible values of heavy metals in rice.

Metal symbol	EWI imported (µg kg^−1^ BW)	EWI Iranian grown (µg kg^−1^ BW)	PTWI^***^ (µg kg^−1^ BW)
Cd	1.69	2.05	7
Pb	7.06	2.52	25
As	0.73	0.59	15
Cr	7.83	3.72	23.3
Zn	44.4	335.34	420
Ni	9.75	2.82	35
Cu	26.69	205.33	500
Co	3.72	3.72	Not set^***^

* ISRI (No: 12968),

**Human body weight,

*** FAO/WHO.

****No PTWI set for Co has been provided there Maximum Tolerable Daily

Intake is (MTDI) value of 0.1 mg kg^−^^1^ body weight (=700 µg kg^−1^ body weight in a week) for this element [5].
