# Supplementary material for: The concentration data of heavy metals in Iranian grown and imported rice and human health hazard assessment
Source: Data Brief. 2017 Nov 21;16:453–9. doi: 10.1016/j.dib.2017.11.057 (PMC5723357; doi:10.1016/j.dib.2017.11.057)
Supplement: Supplementary file 1 — Supplementary material [file mmc1.docx]

**Conflict of Interest**

**The authors of this article declare that they have no conflict of interests**

Ali Jafari^1^, Department of environmental health engineering, School of Health and Nutrition, Lorestan University of medical sciences, Khorramabad, Iran

Bahram kamarehie^1^, Department of environmental health engineering, School of Health and Nutrition, Lorestan University of medical sciences, Khorramabad, Iran

Nahid Khoshnamvand^1^, Department of environmental health engineering, School of Health and Nutrition, Lorestan University of medical sciences, Khorramabad, Iran

Mehdi Birjandi^2^, Department of Biostatistics, School of Health and Nutrition, Lorestan University of medical sciences, Khorramabad, Iran

Mansour Ghaderpoori^1^ Department of environmental health engineering, School of Health and Nutrition, Lorestan University of medical sciences, Khorramabad, Iran
